# Impact of immediate postpartum insertion of TCu380A on the quantity and duration of lochia discharges in Tanzania

**DOI:** 10.1186/s40834-020-00145-2

**Published:** 2021-01-05

**Authors:** Projestine Selestine Muganyizi, Grasiana Festus Kimario, France John Rwegoshora, Ponsian Patrick Paul, Anita Makins

**Affiliations:** 1 Department of Obstetrics & Gynecology, Muhimbili University of Health and Allied Sciences (MUHAS), P.O.Box 7623, Dar es Salaam, Tanzania; 2 FIGO-TAMA PPIUD project, P.O.Box 65222, Dar es Salaam, Tanzania; 3 Obstetrician & Gynecologist, Mbeya Zonal Referral Hospital, P.O.Box 419, Mbeya, Tanzania; 4 FIGO House Suite 3, Waterloo Court, 10 Theed Street, London, SE1 8ST UK

**Keywords:** Postpartum contraception, TCu380A, Pregna, Optima, Lochia discharges, Tanzania

## Abstract

**Background:**

The insertion of Intrauterine Contraceptive Device (PPIUD) for the purpose of contraception immediately after delivery is becoming popular in countries where the use of IUD for contraception has been extremely low. Since 2015, Tanzania implemented the initiative by the International Federation of Gynecology and Obstetrics (FIGO) to institutionalize PPIUD. As a result of capacity building and information delivery under the initiative, there have been increased uptake of the method. Working in this context, the focus of the study was to generate evidence on the effect of TCu380A IUD on amount and duration of lochia and equip service providers with evidence-based knowledge which can help them in counselling their PPIUD clients.

**Objective:**

Establish impact of postpartum TCu380A on amount and duration of lochia.

**Methods:**

A prospective cohort study of delivered women in two teaching hospitals in Tanzania with immediate insertion of TCu380A or without use of postpartum contraception in 2018. TCu380A models; Optima (Injeflex Co. Brazil) and Pregna (Pregna International, Chakan, India) were used. Follow-up was done by weekly calls and examination at 6th week. Lochia was estimated by Likert Scale 0–4 relative to the amount of lochia on the delivery day. An estimated 250 women sample (125 each group) would give 80% power to detect a desired 20% difference in the proportion of women with prolonged lochia discharges among the Exposed and Unexposed groups. Data analysis was by SPSS.

**Results:**

Two hundred sixty women were analysed, 127 Exposed and 133 Unexposed. Medical complaints were reported by 41 (28.9%) Exposed and 37 Unexposed (27.8%), *p* = 0.655. Lack of dryness by end of 6th week was to 31 (23.3%) Exposed and 9 (7.1%) Unexposed, *p* < 0.001. Exposed had higher weekly mean lochia scores throughout with the difference most marked in 5th week (3.556 Versus 2.039, *p* < 0.001) and 6th week (1.44 Versus 0.449, *p*<0.001).

**Conclusion:**

PPIUD is associated with increased amount of lochia and slows progression to dryness within 6 weeks of delivery. The implications of PPIUD clients’ needs to be informed about the possibility of delayed dryness of lochia at time of counseling are discussed.

## Introduction

Intrauterine contraceptive device (IUD) is one of the most effective methods of contraception although in most countries, fewer than a fifth of women rely on this method [1]. Promotion of immediate postpartum IUD (PPIUD) insertion can provide the best option to improve IUD uptake since during this period there is no fear of ongoing pregnancy, causes less discomfort than interval insertion, does not interfere with breastfeeding and can be a one stop method, providing an ample chance for a convenient and cost-effective contraceptive option particularly in settings where women do not return for follow-up visits due to cost or distance [2].

Globally there have been various initiatives to promote postpartum IUD insertion. In 2013, the International Federation of Gynecology and Obstetrics (FIGO) started a large multicountry initiative to institutionalize the practice of antenatal counselling for post partum family planning (PPFP) and training providers on insertion of PPIUD [3]. Under the initiative, more than 36,766 insertions occurred across 48 facilities in six countries: Sri-Lanka, Bangladesh, Nepal, India, Kenya and Tanzania. There was a 52% follow up rate which demonstrated expulsion and removal rates of 2.5 and 3.6% at 6 weeks of follow-up [3]. These complications were lower or comparable with data from systematic reviews and some discrete studies elsewhere [4–7]. One postulated explanantion was that the methodology across all countries under the FIGO initiative was identical, using the Kelly’s forceps to ensure a high fundal placement and TCu380A IUD type as the method of choice.

The postpartum period is complicated by physiological, biological, and emotional changes that could negatively influence contraceptive method continuation. Lochia discharge is one of such physiological changes that hypothetically if excessive or prolonged could lead to IUD expulsion in the same way as excessive uterine bleeding does [4, 8–11]. In support of this connotation it can be observed that the critical period of IUD expulsion coincides with the early postpartum period of 4 to 6 weeks during which lochia discharge is ongoing and uterine involution is taking place [12, 13]. It has been suggested that PPIUD could lead to excessive lochia although there is paucity of research in this area. In one study which compared the duration of lochia discharges among PPIUD clients using Lippes IUD versus mothers without PPIUD, the duration of lochia discharges was on average 31.2 days for PPIUD and 23.3 days for the control with 40% excessive lochia in the PPIUD group compared to 11% in the control group [14]. TCu380A is the most commonly used type of IUD for postpartum contraception, but there is currently scanty literature to support its influence on the amount and duration of lochia discharges. In one follow-up study, 171 women who had undergone PPIUD insertion in India, the rate of abnormal lochia was 10.5% but this was not found to be significantly different from the control goup [15].

The primary objective of this study was to assess the impact of use of TCu380A for immediate PPIUD on amount and duration of lochia discharges compared to non-users of any form of modern contraception. The findings of this study are expected to inform policy, program managers and PPIUD providers with evidence on the impact of TCu380A, when used as an immediate postpartum contraceptive method, on lochia discharges in attempt to improve PPIUD counselling, insertion and method continuation.

## Materials and methods

### The study settings

This is a prospective cohort study of women based in two hospitals in Tanzania – Mbeya Zonal Referral Hospital in Mbeya region which is located in the Southern Highlands of Tanzania and Mount Meru Hospital in Arusha which is located in Northern Highlands. These two hospitals were amongst the six selected for implemention of the FIGO PPIUD Initiative which started in December 2015 in Tanzania**.** The intention was that PPFP counselling and PPIUD services would become routine practice in these hospitals by training of health care providers on postpartum Family Planning counselling, provision of Family Planning Information to clients (health talk, leaflets, posters, and video) as well as Post Partum insertion of TCu380A IUD within 48 h of delivery [3].

The current study hospitals were chosen because they had demonstrated the best turn-up of follow-up clients, had excellent record of PPIUD insertions in the past 2 years and are well equipped with laboratory and ultrasound machines. Since all other implementing hospitals under this project use the same standards in providing PPIUD services, we hoped the study findings emanating from these two study hospitals would be fairly generalizable to other women who receive the services under this program elsewhere in Tanzania.

### The cohort groups

Two cohort groups (Exposed and Unexposed) were identified among women who delivered in the two hospitals between 14th February and 13th May 2018. The Exposed group comprised of women who received PPIUD insertion within 48 h of delivery in the two study hospitals as confirmed by the routine post-partum interviews and procedure documentation. The Unexposed cohort group comprised of women who delivered in same hospitals but did not consent for PPIUD placement or any other form of modern postpartum contraception.

The two cohort groups were matched in terms of time of delivery. The Exposed women were selected from a complete list of clients who had PPIUD placement on that day, often using table of random numbers if there were many eligible women at that time. Once an Exposed woman was recruited and consented to participate, the Unexposed was that woman, who according to delivery records gave birth immediately after the selected Exposed woman on that day. The selected participants in both cohort groups were given information about the study and asked for their written consent to participate.

### Criteria for selection of participants

In order to become eligible for enrolment, a woman fulfilled all of the following: no evidence of chorioamnionitis or purulent vaginal discharge; not ruptured membranes for 18 h or more before delivery; delivered a singleton term pregnancy without instrumentation, delivered in the hospital within 48 h; not developed Post Partum Haemorrhage; has no severe or clinically advanced HIV disease (WHO stage 3 or 4); no condition suspected to distort the uterine cavity such as uterine fibroid; not known to have Wilson’s disease; did not consent for any other longterm method of postpartum family planning except for PPIUD among the Exposed. In the case of two or more women who fulfil the criteria for selection in Unexposed group, a ballotte technique was used to select one among them. Upon agreeing to participate in the study, both the Exposed and the Unexposed were given details about the study and asked to sign a written consent.

### Sample size

A calculated sample of at least 250 women (125 Exposeds and 125 Unexposed) was needed in order to adequately answer the primary objective. This was calculated assuming prolonged lochia discharge beyond 6 weeks of 40% in the Exposed and 20% in the Unexposed group [14, 16], 80% power or greater, a two-sided significance level of 95 and 20% loss to follow.

### Follow-up procedure

Each woman in the study was asked to keep a diary on lochia discharges and was contacted by a Midwife weekly on mobile phone to report on the amount of lochia and any other experienced complications. Illiterate women were asked to keep memory of the lochia flow for a week. Given phone calls were weekly, this was felt to be fairly reliable. During weekly phone calls women were asked about daily amount of lochia from the last physical attendance or call. At the end of the sixth week, all women were given an appointment to attend a clinic for physical examination and the documentation of findings. The information was documented by the attending provider in the follow-up clinic. At this follow-up appointment pelvic Ultrasound was performed on all women whose IUD strings could not be seen in order to ascertain the presence and position of the device. The women in the Exposed cohort group were asked about their experience with the IUD. Blood investigations and vaginal swabs were taken for women who report abnormal lochia in terms of amount, duration, color and smell in both the groups in order to exclude infection.

### Interpretation of key variables

The estimation of amount of lochia was semi-quantitative using a Likert Scale score of 0–4 relative to the amount of lochia discharges experienced on the first day postpartum according to her diary. Thus, the amount of lochia relative to that on the first day was scored as heavier (=Score 4), same (=Score 3) less and moderate (=Score 2), less and scanty (=Score 1), dry (=Score 0). There was no direct measurement of the amount of lochia.

Women who had ongoing lochia discharge at the end of 6th week were considered to have prolonged lochia discharges. Uterine infection was diagnosed if the woman complained or was found on examination to have a foul smelling vaginal discharge plus either increased low abdominal pain/tenderness, fever or positive culture. Method discontinuation was considered to have occurred if: there was voluntary request for removal, fall out of the IUD without replacement, removal due to medical complications or accidental removal without replacement of another IUD by end of 6th week of follow-up. Two models of TCu380A, Optima (Injeflex Co. Brazil) and Pregna (Pregna International, Chakan, India), were in public use in Tanzania. PPIUD providers knew and documented the type of TCu380A Model used as Pregna or Optima but no one was aware of any research interest in these two TCu380A Models. For quality purposes in both hospitals, PPIUD providers were different from Midwives who made weekly phone calls and both these did not conduct the final examination of clients at the end of 6th week.

### Data analysis

Data were downloaded into Excel and exported to Statistical Package for the Social Sciences (SPSS) Version 20 (IBM, Armonk, NY, USA). Descriptive data were compared among the groups using measures of central tendency and proportions. Chi square was used to compare categorical data; means for two groups were compared by T-test if the data were normally distributed and by Mann-Whitney U test if normal distribution was not assumed. One Way Analysis of Varience (ANOVA) was used to compare the varience in means for the analysis of three groups. In all the statistics, a *p*-value < 0.05 was considered significant. Data Safety Monitoring Board (DSMB) was in place in order to ensure all aspects of safety of the FIGO PPIUD Initiative in Tanzania.

## Results

**Fig. 1:**
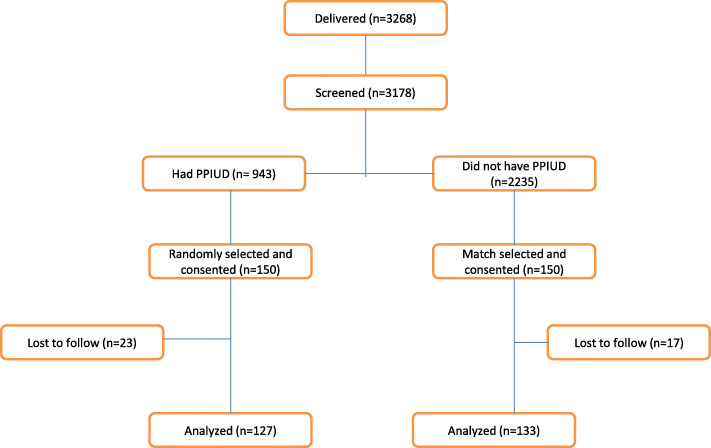
Flow chart

There were 3268 women who delivered in the two study hospitals during the recruitment period from 14th February to 13th May, 2018. Among these, 3178 (97.3%) were interviewed about their PPFP method uptakes of whom 943 (29.7%) had PPIUD insertions. In total 153 women who received PPIUD insertions were eligible for the study but 3 of them did not give consent. Thus, 150 Exposed women who consented for the study were recruited for intensive follow up and implicitly 150 women who delivered immediately after each Exposed woman and consented were selected for the Unexposed cohort group. Among the 300 women who were weekly followed up, 260 (87%) were included in the final analysis at 6 weeks including 127 Exposed and 133 Unexposed (Fig. 1).

Based on Table 1 results, the Exposed and Unexposed groups were comparable at enrolment except for a slightly more Cesarean section deliveries among the Unexposed (27.2%) than the Exposed group (18.3%), *p* = 0.01.

**Table 1:** Characteristics of study participants at enrolment

Characteristics	All	Exposed	Unexposed	Statistics
**Mean Age (±SD)**	26.73 (6.25)	26.73 (6.32)	26.27 (6.19)	0.519^a^
**Delivery Hospital, n (%)**				
Arusha	148 (49.3)	74(49.3)	74(49.3)	
Mbeya	152 (50.7)	76(50.7)	76 (50.7)	0.997^b^
**Highest Education**				
Primary	125 (41.7)	65 (43.3)	62 (41.3)	
Secondary or higher	175 (58.3)	85 (56.7)	88 (58.7	**0.725**^**b**^
**Mean pregnancies (SD)**	2.43(1.78)	2.38(1.69)	2.49(1.86)	0.873^c^
**Mean Ever born children (SD)**	2.36(1.94)	2.38(2.16)	2.33(1.69)	0.944^c^
**Mean Living Children (SD)**	2.14(1.45)	2.09(1.39)	2.19(1.52)	0.613^c^
**Mean Pregnancy loss (SD)**	0.28(1.02)	0.28(1.12)	0.27(0.90)	0.933^c^
**Mode of delivery, n (%)**				
Normal Route	232 (77.3)	125(81.7)	107 (72.8)	0.01
Cesarean Section	68 (22.7)	28 (18.3)	40 (27.2)	
**Breast feeding**	300 (100)	150 (100)	150 (100)	1.0^b^

^a^t-test for equality of Means; ^b^Chi Square test *p*-value; ^c^Mann-Whitney U test

The combined number of women that reported any medical complaint at any time during the 6 weeks’ follow-up was 78 (26.0%) including 41 (28.9%) in the Exposed group and 37 of the 133(27.8%) women in the Unexposed. This difference was not statistically significant (*p* = 0.655). All the medical complaints were minor and 17 (22%) women among the 78 who reported any medical complaint sought medical treatment by contacting a health care provider or buying some medications from a medical store, commonly for pain relieaf (Table 2).

**Table 2:** Frequency of reported medical symptoms among Exposed and Unexposed cohort groups

Medical Condition^**a**^	Exposure status	Week After Delivery						Cummulative Six Weekly Frequency
		1st Wk	2nd Wk	3rd Wk	4th Wk	5th Wk	6th Wk	
Low abdominal Pain	Exposed	12	10	3	4	3	7	39
	Unexposed	16	2	1	1	1	3	24
Abnormal Vaginal discharge	Exposed	4	8	3	2	0	1	18
	Unexposed	6	0	3	3	3	1	16
Fever	Exposed	4	1	0	1	0	0	6
	Unexposed	2	3	1	1	1	0	8
Feeling Unwell (For other reason)	Exposed	4	1	0	0	1	1	7
	Unexposed	6	5	2	2	0	1	16
Vaginal Bleeding	Exposed	0	0	0	0	1	0	1
	Unexposed	0	1	0	0	1	0	2
All Medical conditions	Exposed	24	20	6	7	5	9	71
	Unexposed	30	11	7	7	6	5	66
**Cummulative Weekly Frequency**		54	31	13	14	11	14	137

^a^Multiple responses included

**Fig. 2:**
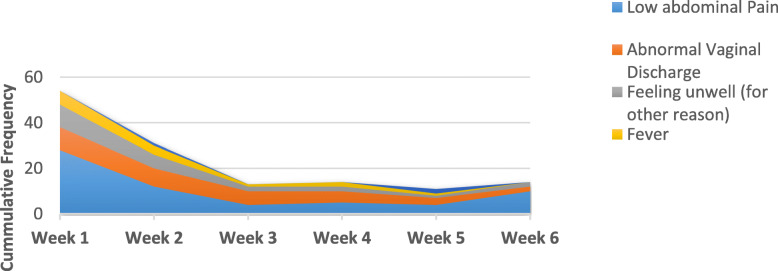
Stacked area chart for frequencies of the reported medical symptoms

The most frequently reported symptoms by the 78 women who reported any medical complaint over the 6-week follow-up were low abdominal pain (reported 63 times) followed by abnormal vaginal discharge (34 times). The least reported was vaginal bleeding (3 times). The pattern of individual medical symptoms reported by the 78 women in the Exposed and Unexposed cohort groups combined is illustrated in Fig. 2.

Most complaints were reported during the first (*n* = 54) and second (*n* = 31) weeks of delivery. The frequency of complaints decreased progressively along the 6 weeks follow-up. In all, there was no confirmed uterine infections.

Overall on the last day of the second week only 62 (22.3%) of all the interviewed participants (*n* = 278) were dry and among them only 28 (10%) were dry for three consecutive days. As time progressed beyond the first week of follow-up, more women experienced intermittent dryness within the same week. On the last day of the fourth week 97 of the interviewed 275 women (=35.3%) were dry but only 58 (21.1%) were dry for 3 days consecutively. At the end of sixth week 220 (84.6%) of the interviewed 260 women were dry with 218 of these being dry for at least 3 consecutive days.

**Fig. 3:**
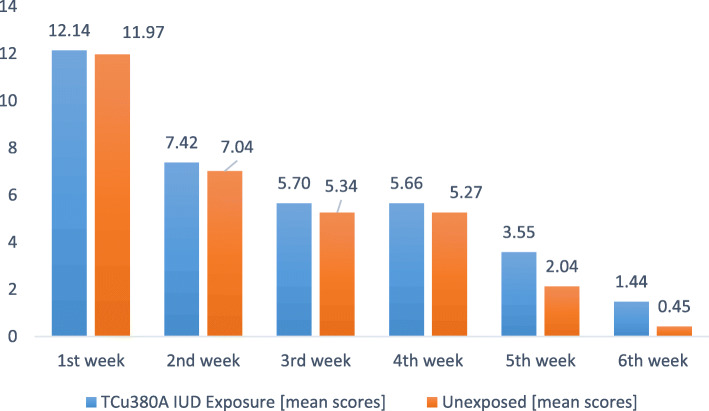
Patterns of mean weekly lochia score among Exposed and Unexposed Cohort groups

Overall 40 (16.4%) among 260 women who were analyzed at the end of their 6th week post-delivery had ongoing lochia discharges including 31 (23.3%) Exposed and 9 (7.1%) Unexposed, *p* < 0.001. The mean weekly lochia scores for the Exposed and Unexposed is illustrated in Fig. 3.

As illustrated in Fig. 3, throughout the 6 weeks of follow-up women in the Exposed group had higher mean lochia scores compared to the Unexposed group. Nevertheless, the illustrated differences in the first week through the fourth week were not statistically significant (*p*<0.05). The difference in mean lochia scores became statistically significant in the 5th week (3.556 Versus 2.039, *p* < 0.001) and 6th week (1.44 Versus 0.449, *p*<0.001).

In a subanalysis of the 133 Exposed women who completed a 6-week followup, 65 used Optima TCu380A (Injeflex Co. Brazil) device model and 68 had Pregna TCu380A (Pregna International, Chakan, India) device model. By the end of the 6th week the mean lochia scores had dropped from 13.08 to 0.382 for Pregna TCu380A and from 11.97 to 0.449 for the Unexposed group. For Optima the change was the least from 11.17 on the first week to 2.72 at the end of 6th week.

**Fig. 4:**
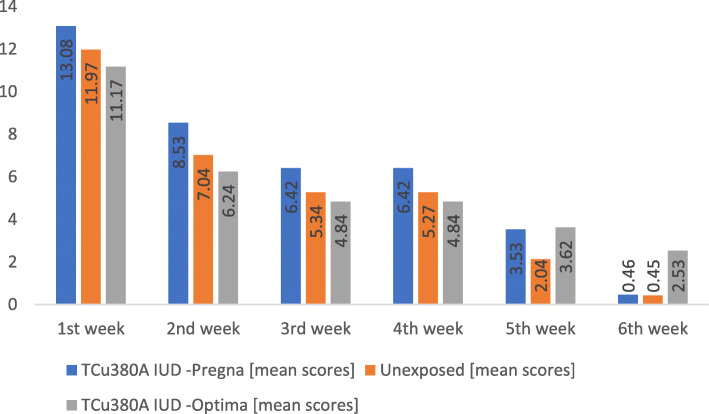
Patterns of weekly mean lochia scores by TCu380A Model and Unexposed Cohort groups

Marked weekly mean lochia score variations were observed for the two TCu380A IUD models used for PPIUD. Pregna was associated with persistently higher weekly lochia scores in the initial 4 weeks compared to the Unexposed group but their progression to dryness by end of 6th week was comparable at the 6th week. Optima was associated with persistently lower weekly lochia scores in the initial 4 weeks but the rate of progression to dryness by the end of 6th week was slowest. All the observed mean weekly lochia score variations among the two TCu380A models and the Unexposed group were compared using ANOVA. Figure 4 illustrates the pattern of weekly lochia scores for the three groups.

The varience in mean weekly lochia scores was statistically significant for each week including F = 11.31, *p* < 0.001; F = 11.57, *p* < 0.001; F = 6.0, *p* = 0.003; F = 6.01, *p* = 0.003 and F = 12.1, *p* < 0.001 and F = 34,65, *p* < 0.001 for the first week through the end of 6th week respectively.

## Discussion

This study was conducted in the context of increasing interest on PPIUD practice globally but with scanty evidence on the effect of IUD on the pattern of postpartum lochia. IUD is available in a variety of shapes and material, but the focus of this study was on TCu380A Model since this was the only Model recommended by the World Health Organization for bulky procurement, hence the approved IUD Model for public service use in Tanzania [17]. The study has shown that minor medical complaints were common and comparable among the Exposed (28.9%) and the Unexposed (27.8%). There was excessive and prolonged lochia discharge with the use of TCu380A immediately postpartum.

The findings in this study support the safety of TCu380A for contraceptive use during the immediate postpartum period. Although a quarter of women who were followed up to completion of their 6th week postpartum developed a variety of medical symptoms, these were minor, mostly self-limiting and comparable among the Exposed and Unexposed women and therefore not attributable to PPIUD. Only one expulsion of the IUD was reported. Altogether, these findings support the safety of postpartum IUD insertion with TCu380A, as has been reported in others studies elsewhere [11–13, 17, 18].

All women in the current study were breastfeeding their babies and by the end of the second week just less than a quarter of them were dry, contrary to the conventional understanding of lochia lasting for 2 weeks [19]. Alternating periods of dry and wet days was the pattern fairly commonly observed between the second and fifth week postpartum although still only a third (34.8%) of them was completely dry for three consecutive days as they entered their fifth week postpartum. Nevertheless, the proportion of postpartum women that continues to be wet by the end of 6th week was about 16% which is comparable with the 11% that has previously been reported by others [15].

This study suggests that TCu380A inserted immediately postpartum affects both the amount and duration of lochia discharges. Exposed women consistently reported more lochia scores throughout the follow-up period compared to the Unexposed, albeit the varience only reaching statistical significe later in the fifth week of follow-up. Likewise, there was evidence that the duration of lochia discharge among women with PPIUD was more prolonged than the Unexposed as indicated by more than a fifth of women in the Exposed cohort group compared with less than a tenth among Unexposed reporting to be wet by the end of 6th week. Previous studies have reported that for women without IUD, the duration of lochia can be altered by Caesarean delivery, prolonged labour and instrumental delivery [20, 21]. Except for Cesarean section, all of these factors were controlled for through case selection criteria according to the study design, hence no impact can be attributed to any of these and the higher Cesarean section rate in the Unexposed group did not seem to affect the amount and duration of lochia. It is unclear yet the mechanism by which TCu380A IUD increases the amount and duration of lochia discharges. The assumption is that this could be related to the ability of TCu380A to evoke inflammatory response and possibly as a foreign body causing delay in endometrial healing and uterine involution [17].

There were two TCu380A Models used by the women in this study. Pregna TCu380A (Pregna International Ltd., India) has been in public use for many years in Tanzania and Optima (Injeflex Company, Brazil) which was more recently introduced for public use in Tanzania at the time of study. The comparison of the two TCu380A was rather exploratory. It was observed that the two could affect lochia discharge substantially differently, with Optima being associated with more prolonged discharge but less in amount compared with Pregna. Apart from minor product finishing differences in the edges of the copper rim on the arms of the T-plastic frames of the two types, we found no other obvious physical differences on the copper frame among the two and the confirmation from the Injeflex Company (direct communication) did not show any substantial additions from those of Optima. The patterns of amount and duration of lochia for the Exposed and Unexposed persisted even on sub-analyses involving cases in each hospital separately. In a previous Cochraine review comparing the removal rates of IUDs no significant differences were observed among framed copper devices, including TCu380A, in terms of IUD removals due to bleeding and pain [11]. Nevertheless, the Cochrane review neither had a focus on TCu380A Model sub-types nor a focus on postpartum IUD insertion complications. Since PPIUD involves keeping the copper device and the coiled threads inside the uterus during the initial 6 weeks it is possible that some variations in thread material and size between Optima and Pregna might have contributed to the observed clinical differences. These differences, however, were not ascertained in this study. Thus, the consistent pattern attributed by the two sub-types of TCu380A was incidental for this study but convincing enough to suggest a randomised controlled trial comparing the two TCu380A Model sub-types.

This study has some important limitations. It was not possible to blind the women on their PPIUD uptake status and the interviewers in each hospital were not blinded about the women’s statuses. These interviewers, however, did not know the details regarding the primary and secondary objectives. Moreover, physical examination on exit were made and documented by different interviewers. Another limitation regarding the study design was the presence of more Cesarean section deliveries among Unexposed than Exposed which could have overestimated the complications for the Unexposed group as post Cesarean section women are more likely to complain of abdominal pain or feeling unwell. However, it is also recognised that vaginal insertions of PPIUD tend to cause more complications than insertions at Cesarean section as the insertion is blind post vaginal delivery [7]. However, these increased complications tend to be related to expulsions or removals which was not the outcome of interest of this study.

The estimation of amount of lochia did not involve direct measurement but rather was scored based on women’s opinion on the amount of lochia relative to the day of delivery. This approach, is potentially disadvantaged in that the exact amount of lochia could not be established. However, we perceived the reliance on woman’s interpretation of the quantity and quality of lochia to be rather a strength in this study since it is the woman’s personal interpretation of a condition that is key for decision to seek medical attention with a consequence of method discontinuation than the reliance on the mere standardized quantities.

This study has contributed to a greater understanding of the effect of the most common copper intrauterine device, TCu380A. WHO and other international organisations recommend that TCu380A IUD should be the preferred device for public-sector procurement on the basis of its efficacy, safety and long history of use [17]. Abnormal lochia discharge may be a common reason for postpartum IUD discontinuation and the early 6 week follow up is critical for the timing of medical removal. Clinicians who see women at 6 week follow-up must be well equipped with proper knowledge on the relevance of increased or prolonged lochia discharges in order to minimize IUD removals, mis-diagnosis of uterine infection and unnecessary treatment with antibiotics. It is also useful to warn women in advance that prolonged lochia can happen in a small proportion of women if PPIUD is the chosen method of contraception.

## Conclusion

TCu 380A IUD when used for immediate postpartum contraception is associated with an increased amount and prolongation of duration of lochia discharges when compared with non-use of any modern postpartum contraceptive method. The currently used model sub-types of TCu380A may have substantially different properties regarding lochia discharges and randomised controlled studies are needed to investigate this effect.

## Data Availability

The datasets used and/or analysed during the current study are available from the corresponding author on reasonable request.

## Abbreviations

ANOVA: One Way Analysis of Varience
DSMB: Data Safety Monitoring Board
FIGO: International Federation of Gynecology and Obstetrics
HIV: Human Immunodeficiency Virus
IUD: Intrauterine Device
MoHCDGEC: Ministry of Health Community Development Gender Elderly and Children
MUHAS: Muhimbili University of Health and Allied Sciences
NIMR: National Institute for Medical Research
PPFP: Postpartum Family Planning
PPIUD: Postpartum Intrauterine Device
SPSS: Statistical Package for the Social Sciences
WHO: World Health Organisation

## References

[1] Winfrey W, Rakesh K (2014). Use of family planning in postpartum period.

[2] Glasier A (2015). Best practice in postpartum family planning.

[3] Canning D, Shah IH, Pearson E, Pradhan E, Karra M, Senderowicz L (2016). Institutionalizing postpartum intrauterine device (IUD) services in Sri Lanka, Tanzania, and Nepal: study protocol for a cluster-randomized stepped-wedge trial. BMC Pregnancy Childbirth.

[4] Chen BA, Reeves MF, Hayes JL, Hohmann HL, Perriera LK, Creinin MD (2010). Postplacental or delayed insertion of the levonorgestrel intrauterine device after vaginal delivery: a randomized controlled trial. Obstet Gynecol.

[5] Grimes DA, Lopez LM, Schulz KF, Van Vliet HA, Stanwood NL (2010). Immediate post-partum insertion of intrauterine devices. Cochrane Database Syst Rev.

[6] Eroglu K, Akkuzu G, Vural G, Dilbaz B, Akin A, Taskin L (2006). Comparison of efficacy and complications of IUD insertion in immediate postplacental/early postpartum period with interval period: 1 year follow-up. Contraception.

[7] Sucak A, Ozcan S, Celen S, Caglar T, Goksu G, Danisman N (2015). Immediate postplacental insertion of a copper intrauterine device: a pilot study to evaluate expulsion rate by mode of delivery. BMC Pregnancy Childbirth.

[8] Chi C, Bapir M, Lee CA, Kadir RA (2010). Puerperal loss (lochia) in women with or without inherited bleeding disorders. Am J Obstet Gynecol.

[9] Fletcher S, Grotegut CA, James AH (2012). Lochia patterns among normal women: a systematic review. J Women's Health (Larchmt).

[10] Visness CM, Kennedy KI, Ramos R (1997). The duration and character of postpartum bleeding among breast-feeding women. Obstet Gynecol.

[11] Kulier R, O'Brien, P., Helmerhorst, F.M., Usher-Patel, M., d'Arcangues, C.: Copper containing, framed intra-uterine devices for contraception. Cochrane Database Syst Rev 2007(4). 10.1002/14651858.CD005347.pub3 PMC12832169 17943851

[12] Goldthwaite LM, Sheeder J, Hyer J, Tocce K, Teal SB (2017). Postplacental intrauterine device expulsion by 12 weeks: a prospective cohort study. Am J Obstet Gynecol.

[13] Shukla M, Qureshi S (2012). Post-placental intrauterine device insertion–a five year experience at a tertiary care Centre in North India. Indian J Med Res.

[14] Hingorani V, Bai U, Kakkar AN (1970). Lochia and menstrual patterns in women with postpartum IUCD insertions. Am J Obstet Gynecol.

[15] Hooda R, Mann S, Nanda S, Gupta A, More H, Bhutani J (2016). Immediate postpartum intrauterine contraceptive device insertions in caesarean and vaginal deliveries: a comparative study of follow-up outcomes. Int J Reprod Med.

[16] Oppenheimer LW, Sherriff EA, Goodman JD, Shah D, James CE (1986). The duration of lochia. Br J Obstet Gynaecol.

[17] Organisation. WH (2018). TCu380A intrauterine contraceptive device (IUD) WHO/UNFPA technical specification and prequalification guidance 2016. In*.* Geneva.

[18] Kumar S, Sethi R, Balasubramaniam S, Charurat E, Lalchandani K, Semba R (2014). Women’s experience with postpartum intrauterine contraceptive device use in India. Reprod Health.

[19] Sonalkar S, Kapp N (2015). Intrauterine device insertion in the postpartum period: a systematic review. Eur J Contracept Reprod Health Care.

[20] Negishi H, Kishida T, Yamada H, Hirayama E, Mikuni M, Fujimoto S (1999). Changes in uterine size after vaginal delivery and cesarean section determined by vaginal sonography in the puerperium. Arch Gynecol Obstet.

[21] Sherman D, Lurie S, Frenkel E, Kurzweil Y, Bukovsky I, Arieli S (1999). Characteristics of normal lochia. Am J Perinatol.

